# Trust under development: The Italian validation of the Epistemic Trust, Mistrust, and Credulity Questionnaire (ETMCQ) for adolescents

**DOI:** 10.1371/journal.pone.0307229

**Published:** 2024-08-26

**Authors:** Alberto Milesi, Marianna Liotti, Francesca Locati, Pietro De Carli, Anna Maria Speranza, Chloe Campbell, Peter Fonagy, Vittorio Lingiardi, Laura Parolin

**Affiliations:** 1 Department of Psychology, University of Milano-Bicocca, Milan, Italy; 2 Department of Dynamic and Clinical Psychology, and Health Studies, Sapienza University of Rome, Rome, Italy; 3 Department of Humanities, University of Pavia, Pavia, Italy; 4 Research Department of Clinical, Educational and Health Psychology, University College London, London, United Kingdom; Medical University of Vienna, AUSTRIA

## Abstract

**Introduction:**

In recent years, the concept of epistemic trust has emerged as a critical factor in understanding psychopathology, particularly within the context of personality disorders. A self-report instrument, the Epistemic Trust, Mistrust, and Credulity Questionnaire (ETMCQ), has demonstrated its validity among English and Italian adult populations. However, extending its applicability to adolescents is essential for comprehending the role of epistemic trust in the development of mental disorders. The aim of this study was to validate the ETMCQ within the Italian adolescent demographic.

**Methods:**

Data were gathered from a wide selection of middle and high schools across Italy. The data collection started on 01/03/2022 and ended on 30/06/2022. Besides the ETMCQ (Study 1 = 662 participants, 12–18 years old, M = 15.56, SD = 2.20; 324 females, 338 males), we also administered other self-report instruments measuring mentalization, emotional dysregulation, general levels of psychopathology, and interpersonal trust in a smaller groups (Study 2 = 417 participants, aged from 12–19 years old, M = 15.64; SD = 2.08; 249 females, 168 males).

**Results:**

Our findings provide empirical validation for the theoretical framework concerning the role of epistemic trust in psychological functioning and substantiate the validity of ETMCQ as a measure to assess it among teenagers.

**Conclusions:**

The ETMCQ is a valid and promising instrument for adolescent populations; its ease and brevity of administration could make it a valuable tool both in clinical and research contexts, shedding light on the role of epistemic trust in mental health.

## Introduction

Recently, epistemic trust (ET) has been introduced as a construct implicated in the development of mentalizing abilities and as a possible vulnerability factor for psychopathology [1–4]. Defined as openness to the consideration of interpersonally transmitted knowledge as reliable and relevant to the self [5], the concept of epistemic trust has been integrated into the framework of mentalization theory. This led to the possibility to consider vulnerability to psychopathology as an outcome of human social cognitive adaptations involving our capacity for and sensitivity to relationally nested cultural communication [3, 6]. Indeed, recent research has highlighted its significant relationships with mentalization and emotion regulation, both of which are fundamental components of adaptive interpersonal and intrapersonal processes [7]. Mentalization, or the capacity to understand and interpret one’s own and others’ mental states, is closely linked to epistemic trust. According to Fonagy and Allison [7], epistemic trust is essential for effective social learning, which relies on the ability to accurately perceive and interpret others’ intentions and emotions. When epistemic trust is established, individuals are more likely to engage in mentalization, as they feel confident in the reliability of the social information they receive. Conversely, a lack of epistemic trust can impair mentalization processes, leading to difficulties in understanding social cues and managing relationships [8]. The relationship between epistemic trust and emotion regulation is equally significant. Emotion regulation involves managing and responding to emotional experiences in a way that is socially appropriate and personally beneficial. Fonagy and colleagues [2] argue that epistemic trust facilitates the internalization of emotion regulation strategies, as individuals who trust their caregivers or therapists are more likely to adopt and effectively utilize the coping mechanisms and regulatory strategies presented to them. This trust is foundational for the therapeutic process, enabling patients to explore and refine their emotional responses with the guidance of a trusted other [1].

Infants are characterized by a natural system of epistemic vigilance that makes them adaptively suspicious toward information coming from others to avoid deceit and harm [9]. This natural state of cautiousness can be reduced by exposure to signals, known as ostensive cues [10], that typically characterize caregiver-baby interactions, such as vocal tone, turn-taking, and eye contact [11–13]. Using these cues, the caregiver signals to the baby that they are reliable, invested and well-intentioned communicators: in other words, these signals trigger epistemic trust and prime the infant to be open to learning [12]. Moreover, caregiver-baby exchanges that reflect the baby’s emotional state, resulting in what authors have called *marked mirroring interactions* fosters children’s mentalization abilities: through marked mirroring, infants can experience themselves as agents characterized by their own mental states, and make the experience of being, in turn, recognized by the other, who can thus be experienced as distinct and separate [4, 8]. Early dyadic experiences that are rich enough in marked mirroring, and which support the relaxation of epistemic trust, generate an expectation in the individual that “a meeting of minds” with others is a positive and adaptive stance; others can therefore be expected to be interesting and interested to think together with [14, 15]. Such expectation, however, may be disrupted by repeated adverse experiences within the caregiving context (i.e., by what has been defined as “complex trauma” [16]): as proposed by Luyten and colleagues [4], complex trauma (concurrently with other factors such as neurobiological and social influences) might be understood as involving profound disruption of the capacity for epistemic trust, which is linked to the possible onset of psychopathology [3].

### Measures of epistemic trust

An initial approach to measuring epistemic trust was made by Orme and colleagues [17] which used a subscale from an existing self-report measure developed to evaluate attachment in adolescence (i.e., the Inventory of Parents and Peer Attachment; IPPA; [18]). In addition, an experimental measure has been developed, which operationalizes ET by assessing the extent to which participants are able to modify their perspective in response to feedback [19]. Campbell and colleagues [5] developed a self-report tool, the Epistemic Trust, Mistrust, and Credulity Questionnaire (ETMCQ), which has been validated in the Italian adult population [20]. The ETMCQ assesses three dimensions: Epistemic Trust (ET), defined as an adaptive stance in which the individual can appropriately learn from social communication, being able to be open to new information; Epistemic Mistrust (EM), defined as the tendency to consider incoming information as untrustworthy and/or not relevant; and Epistemic Credulity (EC), defined as a lack of discrimination in relation to social communications, in which the individual is blindly trustful and prone to manipulation. A recent study by Knapen and colleagues [21] also proposed a new self-report assessment tool for ET.

More generally, studies have shown that the three epistemic stances described may be associated with other dimensions of psychological functioning; both EM and EC are significantly correlated with the presence of childhood traumatic experiences, attachment avoidance and anxiety, low mentalization abilities, difficulties in emotional identification, expression, and regulation, as well as higher levels of psychopathological symptoms [5, 20].

However, extant data relates to adult samples, and although some studies have been conducted on children [22, 23], empirical investigations relating to ET in adolescence were conducted before the development of the ETMCQ [17, 24]. Since epistemic trust has significant relevance in the construction of socio-relational functioning [3, 25], and given its relevance in the development of personality disorders—which often emerges in adolescence [26, 27]—having a tool to easily assess it in teenage years seems crucial to pave the way for further investigations regarding its role as a risk factor for psychopathology. During adolescence, the individual undergoes several changes, especially in perspective-taking, emotion regulation, executive functioning, risk-taking, and identity [28–30]. Teenagers face major developmental tasks, such as mentalizing a transforming body [31, 32], building a stable identity [33, 34], and rearranging attachment relationships [35, 36]. Being able to learn from others, both parents and peers, is central in this life phase [33, 37]. Indeed, it is through relational exchanges that adolescents learn how to regulate their emotions—which are, during this life phase, often characterized by high degrees of intensity [38]. Different studies (e.g., [38–40]) have highlighted the association between mentalization, emotion dysregulation, and internalizing and/or externalizing problems in teenage years. How these dimensions interact with epistemic trust in adolescence was the subject of the current study, which sought to validate the ETMCQ in an Italian adolescent population.

### Objectives and hypotheses

Our hypotheses were threefold:

#### Study 1

A) examine the instrument’s factorial structure through Confirmatory Factor Analysis; B) assess disparities in ET, EM, and EC levels between genders (previously explored in UK and Italian validations with adult samples [5, 18]), alongside exploring correlations between these epistemic stances and age;

#### Study 2

explore the connections between ET, EM, and EC and other psychological variables such as reflective functioning, emotion dysregulation, and psychopathology.

## Methods

### Participants

Data were collected in collaboration with various middle and high schools in Italy. The data collection started on 01/03/2022 and ended on 30/06/2022. Schools were randomly selected throughout the whole country, contacting school boards to request their participation. If the school agreed, principals and teachers informed parents and students about the research to obtain informed consent, clearly stating that the participation of each scholar would be voluntary. The total sample (Study 1) was composed of 662 adolescents aged 12 to 18 years (M = 15.56, SD = 2.20; 324 females, 48.9%; 338 males, 51.1%). All participants were Italian. In the global sample (Study 1), 141 adolescents were attending middle school (21.3%), while the remaining 521 were attending high school (78.7%). The subgroup involved in Study 2 was composed of 417 adolescents aged from 12 to 19 years old (M = 15.64; SD = 2.08; 249 females, 57.5%, 168 males 42.5%). In the Study 2 sample, 77 adolescents were attending middle school (18.5%), while the remaining 340 were attending high school (81.5%).

The inclusion criteria for this study were as follows: being aged from 12 to 19; being Italian speaking; having no intellectual disability or neuropsychiatric disorders, which were screened for by asking caregivers to complete the *Child Behaviour Checklist 6–18 Version* (CBCL; [41]) before adolescents participated in the study. Also, the presence of diagnoses of neuropsychiatric disorders and intellectual disabilities was assessed through parental reports. Thresholds for establishing the presence of psychopathological conditions were determined based on the existing literature on CBCL clinical cut-offs [42]. However, no participants were excluded from the sample. This study’s design and its analysis were not pre-registered.

Data were collected online from March 2022 to June 2022. Parents were informed of the study’s objectives and procedures through virtual meetings. Written parental consent was required for participation. After this, adolescents were provided with information about the study. If they agreed to participate, they were asked to provide informed consent via electronic acceptance of study materials and then redirected to Qualtrics (www.qualtrics.com) to complete the survey. Each assessment session task took 30 to 40 minutes. Tasks were administered through four different links to avoid fatigue. No financial incentive or compensation for participation was provided. The study protocol (Protocol N. t. 0099187/21) was approved by the Ethics Committee of the University [*masked for blind review]*.

All data are publicly available in the supplemental material section of this paper.

### Measures

#### Epistemic Trust Mistrust Credulity Questionnaire (ETMCQ; [5])

The ETMCQ is a 15-item questionnaire in which responses are rated across a 7-point Likert scale (from 1 = “strongly disagree” to 7 = “strongly agree”). The instrument permits the assessment of three dimensions: Trust, Mistrust, and Credulity. An example of a Trust item is: “I find information easier to trust and absorb when it comes from someone who knows me well”. An example of a Mistrust item is: “If you put too much faith in what people tell you, you are likely to get hurt”. A Credulity item is: “When I speak to different people, I find myself easily persuaded even if it is not what I believed before”. The tool has shown good psychometric properties both in the English and Italian validation on the adult population. Cronbach’s α were .70, .65, and .81, respectively [5], and .72, .67, and .73 [20].

#### Reflective Functioning Questionnaire for Youth (RFQ-Y; [43])

The RFQ-Y is a self-report tool for the assessment of reflective functioning (i.e., mentalization) abilities. It contains 46 items, which are rated on a 6-point Likert scale (from 1 = “strongly disagree” to 6 = “strongly agree”). An overall score is obtained by summing recorded responses, with high scores representing higher reflective functioning abilities. In adolescent samples, the RFQ-Y has shown promising psychometric properties. Ha et al. [44] found that the RFQ-Y exhibited good internal consistency (Certainty: α = .78; Uncertainty: α = .81) and construct validity, correlating well with related constructs such as empathy and emotion regulation. Factor analysis supported the two-factor model in this demographic [44]. The questionnaire was translated into Italian by our team and then backtranslated.

#### Inventory of Parent and Peer Attachment (IPPA; [18])

The IPPA is a 75-item self-report assessment tool for the perceived quality of attachment relationships with mother, father, and peers. Items are rated on a 5-point Likert scale, ranging from 1  =  “almost never or never true” to 5  =   “almost always or always true”. Besides an overall score, the tool allows the assessment of three dimensions: communication, alienation, and trust (regarding the mother, the father, and peers). The three IPPA trust subscales were selected to obtain a measure for convergent validity with the ETMCQ. Even though the conceptualization of trust underlying the IPPA trust subscales is broader than epistemic trust—a construct focused specifically on the ability to consider interpersonally transmitted knowledge as both reliable and relevant to the self—we assumed that individuals presenting disruption in epistemic trust would be likely to score low on the three IPPA trust scales. In adolescent populations, the IPPA has demonstrated robust psychometric properties. The scale shows high internal consistency, with Cronbach’s alpha coefficients typically exceeding .80 for all subscales [18] For instance, Gullone and Robinson [45] reported alpha values ranging from .86 to .91 for the parent attachment scales and from .86 to .88 for the peer attachment scale in a sample of adolescents. Test-retest reliability over a three-week period was also strong, with correlation coefficients ranging from .93 to .95 [18].

*Difficulties in Emotion Regulation Scale* (DERS; [46]) is a 36-item self-report assessment measure for the evaluation of difficulties in emotion regulation (ER), with a particular emphasis on negative emotions. Items are rated on a 5-point Likert scale, ranging from 1 = “almost never” to 5 “almost always”, with high scores indicating more difficulty in affective modulation. The instruments assess six domains: lack of emotional awareness, lack of emotional clarity, difficulties controlling impulsive behaviors when distressed, difficulties engaging in goal-directed behavior when distressed, nonacceptance of negative emotional responses, and limited access to emotion regulation strategies. A total score can be obtained through the sum of the score in each subscale. In adolescent populations, the DERS has demonstrated good psychometric properties. For instance, Neumann et al. [47] reported satisfactory internal consistency (α = .89) and test-retest reliability (r = .88) in a sample of adolescents. Moreover, confirmatory factor analyses have supported the six-factor structure in this age group [48]

#### Youth Self-Report (YSR; [49]

The YSR is a self-report questionnaire consisting of 112 items, developed to assess problems in children and adolescents aged between 11 and 18. Items are rated on a 3-points Likert scale ranging from 0 = “not true” to 2 = “very true or often true”. The instrument consists of eight subscales (anxious/depressed, withdrawn/depressed, somatic complaints, rule-breaking behavior, aggressive behavior, social problems, thought problems, and attention problems). Summed together, three of them (anxious/depressed, withdrawn/depressed, somatic complaints) provide a measure of internalizing symptoms, two (rule-breaking behavior, aggressive behavior) of externalizing ones; the last three (social problems, thought problems, and attention problems) are not assigned to any second-order domain. A total score is obtained by summing the scales to garner assessment of the subject’s general factor of psychopathology [50]. The YSR has been extensively validated across diverse adolescent populations. Achenbach et al. [49] reported high internal consistency for the YSR scales (mean α = .84) and strong test-retest reliability (r = .79). Moreover, the YSR’s factor structure has been replicated in various studies, confirming its applicability and reliability in adolescent samples [49]

### Data analysis

Statistical analyses were performed using RStudio version 2022.07.2 and SPSS software version 28.0.1.0. R software was used for the Confirmatory Factor Analysis (CFA) using the lavaan package(v. 0.6–12) and *semPlot* (v.1.1.6) packages. Descriptive statistics, independent sample t-test, and zero order correlations were performed with SPSS software. Data were tested for normality (skewness and kurtosis). Significance was attested when *p* ≤ 0.05 in all analyses.

ETMCQ-A psychometric properties were tested as it follows:

1. **CFA** allowed us to test the original structure found in the English version of the questionnaire for adults [5]. Normalized mean and covariance residuals were found satisfactory. Considering item distribution, Diagonally Weighted Least Squares (DWLS) was used as an estimation method and robust indexes were considered.

2. Model fit was assessed using two relative indices: the robust Comparative Fit Index (CFI) and the robust Tucker-Lewis Index (TLI). Also, two absolute indices of overall model fit were evaluated: robust Root Mean Square Error of Approximation (RMSEA) and robust Standardized Root Mean Residual (SRMR). The thresholds for these indices, according to Kline’s guidelines [46], were defined as follows: CFI and TLI with values ≥ .95 indicating a good fit, and values ≥ .90 indicating an adequate one; RMSEA with values <0.05 indicating an excellent model fit, values between 0.05–0.08 moderate fit, and values between 0.08–0.10 acceptable fit; SRMR with values between 0.05–0.08 indicating an acceptable fit.

3. **Independent sample t-test** was conducted to explore the variance in mean values across dimensions between individuals categorized by their birth-assigned sexes as male and female.

4. **Pearson’s correlation coefficient** was examined to explore the connections between epistemic trust, mistrust, credulity, age, and additional variables under investigation.

## Results

### Study 1

As shown in Table 1, the CFA confirmed the three-dimensions model and all the fit statistics provided acceptable values. All items had substantial and significant loadings in the expected direction on their respective factors, except for Item 3 that is just below the lowest acceptable score of .30. However, an additional analysis suggested no substantial changes in the indexes of the CFA following the omission of Item 3. Therefore, the full model has been preferred since it allows more consistency with the original instrument.

**Table 1:** Fit measures.

					RMSEA 90% CI	
χ^2^	CFI	TLI	SRMR	RMSEA	Lower	Upper
242.48*	.941	.930	.062	.058	.051	.064

Note: Comparative Fit Index (CFI); Tucker-Lewis Index (TLI); Standardized Root Mean Square Residual (SRMR); Root Mean Square Error of Approximation (RMSEA)

*p < .001.

**Fig 1:**
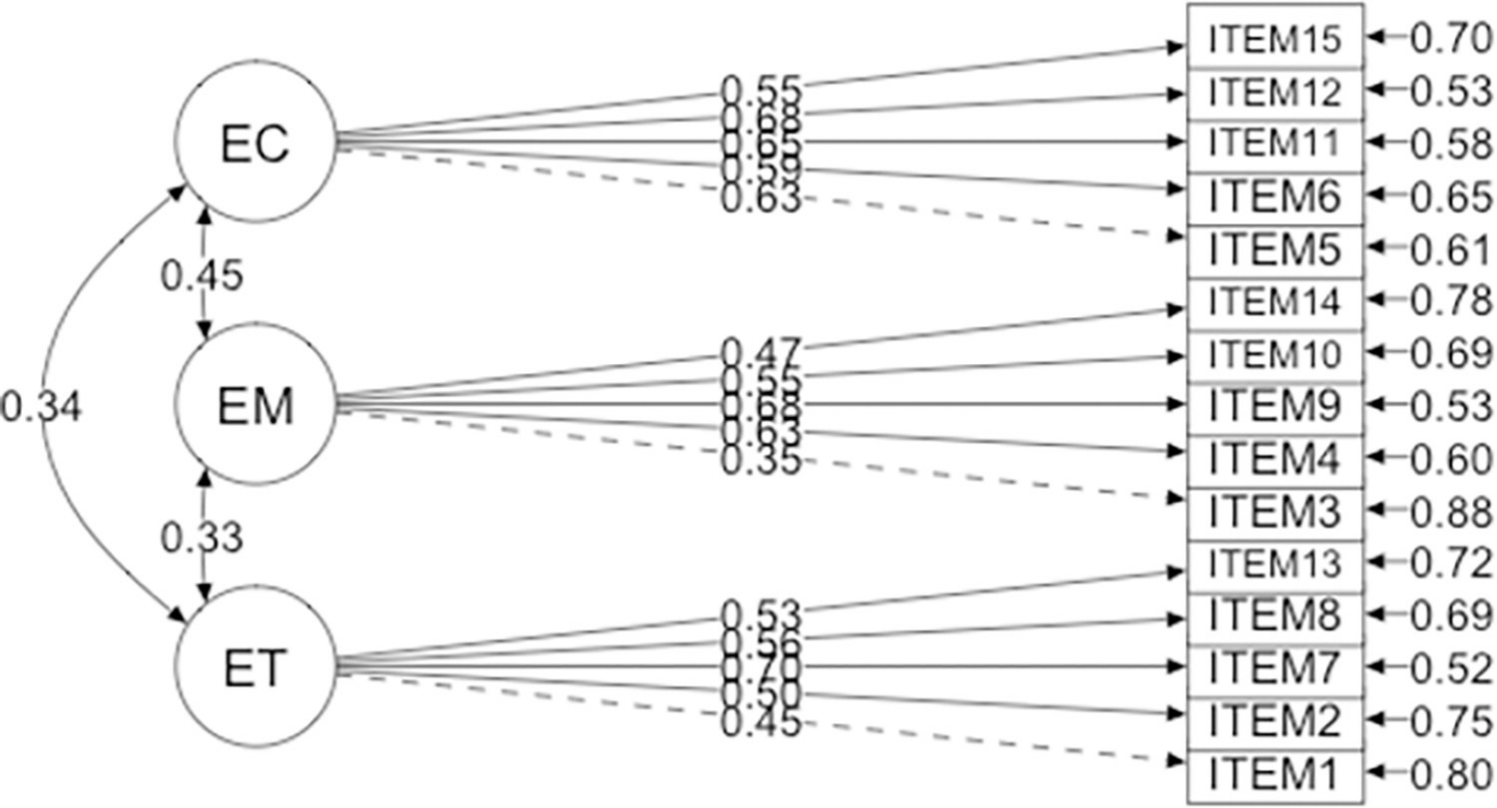
CFA with factor loadings from Study 1. Note: Trust = Epistemic Trust; Mistrust = Epistemic Mistrust; Credulity = Epistemic Credulity.

ET showed significant positive associations with both EM and EC. At the same time, EM was positively associated with EC. In Fig 1 the numbers on the arrows between the three factors represents Pearson r’s coefficient (*p* < .001).

Ultimately, the internal consistency of the three scales was evaluated using Cronbach’s alpha, demonstrating acceptable values: ETα = 0.679; EMα = 0.671; ECα = 0.748.

#### Correlations with demographic variables

ET means in the female group (M = 4.83; SD = .96) significantly differed from ET means in the male group (M = 4.55; SD = 1.15), *t*(660) = -3.43, *p* < .001. EM means in the female group (M = 4.88; SD = .94) also differed significantly from EM means in the male group (M = 4.17; SD = 1.02), *t*(660) = -9.41, *p* < .001. Similarly, among females EC mean scores (M = 3.85; SD = 1.27) were significantly different than EC means in the male group (M = 3.14; SD = 1.16), *t*(660) = -7.44, *p* < .001. A specific caution should be considered regarding gender differences since partial measurement invariance (Δχ2(12) = 16.39, p = .17) but not full invariance (Δχ2(12) = 43.47, p < .001) were supported.

Age was weakly and positively associated only with ET, *r*(660) = .08, p < .05. It showed non-significant association with EM, *r*(660) = .04, *p* = .27, nor EC, *r*(660) = -.02, *p* = .62.

### Study 2

#### Convergent and divergent validity

Means, standard deviations, and zero-order correlations for each variable included in the study are shown in Table 2 and Table 3.

**Table 2:** Mean (M) and Standard Deviations (SD) of the variables studied.

	M	SD
**ET**	4.82	1.03
**EM**	4.57	1.04
**EC**	3.50	1.03
**RF**	6.30	0.64
**DERS_TS**	83.01	24.47
**YSR_TS**	53.02	25.88
**IPPA_FT**	36.28	6.11
**IPPA_MT**	38.28	5.73
**IPPA_PT**	38.03	7.04

*Note*: *Epistemic Trust (ET)*, *Epistemic Mistrust (EM)*, *Epistemic Credulity (EC)*, *Reflective Functioning (RF*), *Difficulties in Emotion Regulation Scale Total Score (*DERS_TS*)*, *Youth Self-Report Total Score (*YSR_TS*)*, *Inventory of Parents and Peers Attachment Father trust (IPPA_FT)*, *Inventory of Parents and Peers Attachment Mother trust (IPPA_MT)*, *Inventory of Parents and Peers Attachment Peers trust (IPPA_PT)*.

**Table 3:** Correlations between the main variables of the study.

	ET	EM	EC
**RF**	.37**	.01	.02
**DERS_TS**	-.01	.48**	.42**
**YSR_TS**	-.06	.50**	.38**
**IPPA_FT**	.11*	-.16**	-.08
**IPPA_MT**	.14*	-.20**	-.11*
**IPPA_PT**	.38**	-.13**	-.15**

*Note*: *Epistemic Trust (ET)*, *Epistemic Mistrust (EM)*, *Epistemic Credulity (EC)*, *Reflective Functioning (RF)*, *Difficulties in Emotion Regulation Scale Total Score (*DERS_TS*)*, *Youth Self-Report Total Score (*YSR_TS*)*, *Inventory of Parents and Peers Attachment Father trust (IPPA_FT)*, *Inventory of Parents and Peers Attachment Mother trust (IPPA_MT)*, *Inventory of Parents and Peers Attachment Peers trust (IPPA_PT)*.

** p < .001

* *p* < .05.

Various positive and negative correlations were found between the three epistemic dimensions (ET, EM, and EC) and the other variables studied. Specifically, ET was positively associated with reflective functioning (RF), trust towards the father (IPPA_FT), trust towards the mother (IPPA_MT), and trust towards peers (IPPA_PT). EM was positively associated with emotional dysregulation (DERS_TS) and psychopathology (YRS_TS) and negatively associated with trust towards the father (IPPA_FT), trust towards the mother (IPPA_MT), and trust towards peers (IPPA_PT). Last, EC was positively associated with emotional dysregulation (DERS_TS) and psychopathology (YRS_TS), and negatively associated with trust towards the mother (IPPA_MT) and trust towards peers (IPPA_PT).

## Discussion

### Study 1

The primary aim of the present study was the validation of the ETMCQ in an adolescent Italian sample. Findings from the CFA confirmed the model proposed by Campbell and colleagues [5] and empirically corroborated in the original validation of the instrument on the English and Italian adult population [5, 20]. All items loaded in the expected direction and on their respective factors, namely: Trust (the capacity to regard interpersonally transmitted knowledge as reliable and relevant for the self), Mistrust (the propensity to systematically regard such knowledge as untrustworthy, thus remaining wary during social interactions), and Credulity (the tendency to show low levels of epistemic vigilance and, therefore, to be more vulnerable to deception and misinformation). In contrast with other findings [5, 20], the three factors correlated positively one with another. It is worth noting that the Cronbach’s alpha values for the EM and ET scales were, albeit acceptable, rather low (i.e., around 0.7). However, our findings are consistent with previous validation and empirical studies, underscoring a recurring challenge in the measurement of these constructs [51, 52]. Finally, the positive correlations among ET, EM and EC subscales are intriguing and warrant further exploration. They suggest that the three epistemic stances, while distinct, may not be entirely independent of one another. This interrelation can be interpreted through several lenses. First, adolescents may exhibit a complex interplay of trust, mistrust, and credulity in their social interactions, reflective of the dynamic nature of epistemic development during this life phase. For instance, a teenager might generally trust information coming from parents, but also exhibit hypertrophic skepticism in specific contexts (e.g., with authority figures such as teachers), and/or show gullibility when under peer pressure. Moreover, during adolescence individuals are in a “transitional” space, one in which their emotional, cognitive and social abilities are still evolving. This period could therefore easily be marked by fluctuations and inconsistencies in how they perceive and evaluate social information: the positive correlation between ET, EM, and EC subscale may thus reflect the ongoing process characterizing these years.

Our second aim was to test the mean differences in ET, EM, and EC levels between males and females, as well as the correlations between the three different epistemic stances and age. Results showed that, among adolescents, gender seems to have a significant effect on all three epistemic positions. The female group presented higher scores on all factors; we found that they had a greater tendency to trust information coming from others than males, but also a higher propensity to be more gullible and (in an apparent paradox) more skeptical and suspicious. These results may reflect a general tendency among females to declare higher levels of agreement: research has already shown that female teenagers possess higher levels of openness and agreeableness than males [53]. Neuroimaging studies using trust games have shown that there are no gender differences in baseline trust among teenagers [54]; the statistical difference between the two gender identities investigated in this study may thus be a product of the use of self-report assessments rather than indicative of an actual difference in ET, EM, or EC. Moreover, in the Italian validation of the ETMCQ on the adult population [20], no effect for gender was found in any of the three epistemic stances, suggesting that the tendency described above is characteristic of the teenage population, at least in Italian culture. In relation to the association between the three epistemic stances and age, we found a small positive correlation between age and ET, but non-significant associations between EM or EC and age. The ability to adaptively trust information transmitted through social exchanges seems therefore to increase with age, corroborating findings from other studies [55, 56] and congruent with the evidence that social cognitive abilities develop across adolescence and in early adulthood [57, 58]. This result seems particularly relevant considering that higher levels of trust are also associated with greater psychological well-being and perceived self-efficacy [5, 55, 59]. This finding is in line with that of the validation of the ETMCQ on the Italian adult population [20], where a negative correlation between mistrust and age was reported.

### Study 2

Our third aim was to explore the associations between ET, EM, EC, and different facets of psychological functioning, such as mentalizing capacity, emotional regulation, general levels of psychopathology, and—to obtain a measure of convergent validity—levels of trust towards caregivers and peers. Our findings supported the hypotheses of the study; namely, the three epistemic stances possess distinct and statistically meaningful correlations with the dimensions considered in our analyses. All such correlations were in the expected direction, corroborating theoretical suggestions that epistemic disruptions may constitute an underpinning vulnerability factor for psychopathology [2, 4], and further substantiating empirical contributions about epistemic trust [5, 20]. On the question of convergent validity, the three ETMCQ factors showed significant, although modest, correlations with the three trust subscales of the IPPA, indicating that epistemic trust represents a related but distinct construct, able to broaden the perspective on psychological functioning and to offer a unique perspective.

More specifically, ET showed a significant positive association with all three subscales (regarding trust in father, mother, and peers) of the IPPA. The correlations between ET and trust in the maternal and paternal figure were quite small; the higher one was between ET and trust in peers, showing a moderate magnitude. This is an intriguing finding. It may reflect one of the active tasks that adolescents are engaging in: actively expanding they constituency for epistemic trust beyond the home environment. These results are in line with what has been described in the literature: during adolescence, individuals become increasingly independent from their family systems and peers start to play a more crucial role within the social network, as well as to represent influential sources of support (for a review, see [60]). ET showed also a significant moderate correlation with reflective functioning, corroborating the hypothesis that the ability to reflect on one’s own and others’ mental states is closely associated with the ability to learn new things about—and *through*—the interpersonal world. Especially if present during the teenage years, such ability could promote flexibility in considering what happens at both an intrapsychic and social level, thus a better ability to understand it in terms of mental states. However, further studies are needed to better investigate if, and how, mentalization stances and epistemic trust develop and influence one another, especially during teenage years. Finally, it is important to note that we found no significant association between ET and youth psychopathology (as assessed by the YSR). This finding aligns with recent studies that have questioned the utility of the ET subscale in nonclinical/community samples. For instance, Asgarizadeh & Ghanbari [51] reported lower reliability and discriminant validity for the ET subscale in nonclinical populations compared to clinical settings, suggesting limited applicability in everyday social interactions within community samples. Similarly, Asgarizadeh, Hunjani, & Ghanbari [61] found that the ET subscale did not add significant predictive value over epistemic mistrust and credulity in predicting mentalizing-related constructs in nonclinical populations. Additionally, in the original ETMCQ validation study on the UK population, Campbell and colleagues [5] noted that the ET scale was not significantly associated with lower mental health symptoms, nor did it function as a moderator in mitigating the effects of childhood adversity, indicating that more than acting as a protective or resilience factor, ET might be better conceptualized as a basic mode of social functioning. These findings suggest that the role of epistemic trust in nonclinical settings may be more complex and context-dependent than previously thought.

EM, instead, showed small negative correlations with all three IPPA trust subscales, with similar effect sizes for each subscale. Thus, when adolescents show high levels of epistemic mistrust, which involves a wary and rigid attitude towards socially transmitted knowledge, they tend to hold negative perceptions towards their relationship with all their significant others. This result seems to substantiate the theoretical position according to which excessive epistemic vigilance—which is often the result of early adverse interpersonal experiences—can lead the individual to a sort of “epistemic petrification”. In this state, others are perceived as not reliable or trustworthy, leading not only to a reduced capacity to learn from and adapt to the social, but also generating a vicious relational cycle in which negative beliefs about the trustiness and dependability of others are confirmed. This, in turn, might produce an intolerable sense of isolation in the mistrustful individual, and the perception that such an individual is “hard to reach” in others [1]. It is this closing off from the possibility of joining with other minds that explains why EM has been posited as a risk factor for the development and maintenance of psychological suffering [3]. The results presented here support this hypothesis: EM showed moderate-to-high correlation with both higher difficulties in emotional regulation and higher levels of internalizing and externalizing symptomatology. Several previous studies [38, 39, 62–65] have shown that the ability to recognize and regulate one’s emotions is a key factor in wellbeing and, when lacking, constitutes a risk factor for the development of psychopathological conditions. This appears to be particularly the case during adolescence, a period characterized by rapid and often difficult changes, frequently accompanied by tumultuous emotions. In this life phase, the inflexible wariness that characterizes EM seems to be associated not only with emotional dysregulation but also with a greater vulnerability to a range of psychological symptoms, as shown by the correlation between epistemic mistrust and the overall score at the YRS.

Finally, we found small but negative correlations between EC and two of the IPPA subscales, those relating to trust in the maternal figure and in peers. These results suggest that adolescents’ perception of their relationships as characterized by mutual support and acceptance is compromised not only when they present high mistrust, but also (albeit less considerably) when they have a maladaptive tendency to overly trust information coming from others. The absence of a significant correlation between EC and trust in the father figure as measured by the IPPA represents an interesting finding. It is possible to speculate that a credulous epistemic attitude in adolescent years—a stage of life characterized by an urge to reduce one’s dependency on caregiving figures and find autonomy (i.e., by separation-individuation)—leads to ambivalent feelings towards a more authoritative figure, such as the paternal one. As for the association between epistemic credulity and other measures of psychological findings, our results show that, similarly to EM, EC shows moderate correlation with both higher difficulties in emotional regulation and higher levels of internalizing and externalizing symptoms. Previous contributions [4, 66] have proposed that credulity might be linked to a hypertrophic, unmoored imaginative activity lacking high-level processing, and showed how this is often the consequence of complex trauma [67–71]. This could result in a compromised ability to mentalize internal self-states and thus to self-regulate, as well as a reduced capacity to recognize a divergence between self-perception and others’ perception of the self, which has previously been described as an “epistemic mismatch”. Such process could lead adolescents to be more vulnerable to exploitation or misinformation, passively accepting others’ interpretations and explanations. Moreover, given the ever-changing intra- and inter- personal context in which adolescents are immersed, subsequent discordances between their experience and the information assimilated through the social environment could make credulous teenagers more at risk of increased disappointment/sense of betrayal, alienation, and inability to be understood and accepted, thus making them more vulnerable to develop psychopathological symptoms, as the positive correlation with the YRS found in this study seems to suggest. Last, both EM and EC did not show significant associations with reflective functioning, in contrast to existing literature [5]. This may be due to the adoption of a different measure for the assessment of reflective functioning: both Campbell and colleagues [5] and Liotti and colleagues [20] included the Reflective Functioning Questionnaire [72], while in this study we used the Reflective Functioning Questionnaire for Youth (RFQ-Y; [43]) which is a slightly different assessment measure of mentalization. The items of the RFQ-Y may capture different aspects of reflective functioning that are less “sensitive” to the disruptions caused by EM and EC. On the other hand, it seems that EM and EC lead teenagers to show more unpredictable and inconsistent reflective processes, although not yet fully compromising their mentalization abilities. This was an unexpected finding, highlighting the need for further investigation into the distinct pathways through which epistemic trust and its disruptions may influence mentalizing abilities over the course of development. Additionally, it seems necessary to more thoroughly examine the role of contextual factors such as peer relationships, family dynamics, and socio-cultural influences.

In conclusion, the associations between ET, EM, EC, and the psychological dimensions explored in this study appear to offer a contribution to our understanding of adolescent psychopathology as well as risk and resilience factors, both in psychotherapy and in the broader social environment. However, further studies are needed to understand more fully how and to what extent epistemic trust and its disruption might be implicated in the development of psychopathology.

## Limitations

Even though the results of this study corroborated all our initial hypotheses, there are some limitations that should be considered. First, we did not include a test-retest measure in the study. Further research is necessary to determine the consistency of ETMCQ results over short periods of time. Moreover, we only used self-report measures, which—although presenting some clear advantages—can introduce some bias (for example, as mentioned in the discussion, adolescent females might show an “acquiescence response style”). It is also crucial to consider that maladaptive or traumatic interpersonal contexts could significantly impact the development of ET. Such contexts may contribute to the variability in these constructs, further complicating their measurement and interpretation, especially during developmental age. Future studies should more carefully consider the impact of adverse interpersonal contexts on the development of these constructs, employing a more nuanced approach to better capture the complex nature of this construct. It would be fruitful to combine self-report and behavioral assessments to test the presence of eventual discrepancies between self-perceptions in epistemic trust and actual social conduct. Unfortunately, no precautions were taken during data collection to prevent careless responding. Despite a mechanical statistical procedure implemented post hoc to control for potentially careless responders substantially confirmed the results, we cannot exclude that low attention in participants could affect the study results. Moreover, we only investigated the convergent validity of the instrument, measuring the correlation between the three epistemic stances and related concepts, such as trust in significant others or reflective functioning. Further studies could focus on assessing the discriminant validity of the instrument, to more fully test this novel construct. Another limitation resides in the fact that our study’s approach to gender is limited by a binary classification, which does not capture the full spectrum of gender identities, especially among adolescents today. This dichotomous approach may overlook important nuances and variations in the data. Future research should incorporate a more dimensional assessment of gender to better understand if, and how, different gender identities can have an influence on epistemic trust, mistrust, and credulity.

## Conclusion

Our findings indicate that the ETMCQ—the first self-report tool for the measurement of epistemic trust, mistrust, and credulity—represents a valid and promising instrument for adolescent populations. Its ease and brevity of administration could prove to be valuable in both clinical and research contexts. Since theoretical contributions have postulated that epistemic disruption may represents a vital factor in explaining not only how psychopathology develops, but how it is maintained through a lack of the ability of feeling understood by others and of using interpersonal exchanges as a source of information both reliable and relevant to the self. Having a tool for rapidly assessing ET, EM, and EC in teenagers—and their interplay with other psychological dimensions—could substantiate what has already been suggested regarding their fundamental role in adolescent psychopathology [14, 17, 24, 73] and in the building of a therapeutic relationship able to promote a cooperative, attuned and mentalizing stance on both participants of the dyad, all factors that promote adaptive and durable changes [74–76], and seem to play a fundamental role during teenage years [77, 78]. Moreover, the ETMCQ could be a valuable tool not only in research about psychopathology and psychotherapeutic processes: indeed, epistemic trust has already proven to be an effective concept in explaining other and broader aspects of our cultural and social world. Research has shown that it can help us understand phenomena such as belief in conspiracy theories or fake news [79, 80], attitudes toward climate change [81], and vaccine hesitancy [82, 83].

## Supporting information

S1 File Study dataset. Full dataset used in the study.
(XLSX)
